# Sessile Serrated Lesion Detection Rate and Colorectal Cancer Risk and Mortality

**DOI:** 10.1001/jamanetworkopen.2025.56964

**Published:** 2026-02-17

**Authors:** Edward S. Huang, Qiwen Huang, Pragati Kenkare, Satish Mudiganti, Meghan C. Martinez, Scarlett Lin Gomez, Su-Ying Liang

**Affiliations:** 1 Department of Gastroenterology, Palo Alto Medical Foundation, Sutter Health, San Jose, California; 2 Center for Health Systems Research, Sutter Health, Palo Alto, California; 3 Center for Health Systems Research, Sutter Health, Walnut Creek, California; 4 Department of Epidemiology and Biostatistics, University of California, San Francisco

## Abstract

**Importance:**

Although the adenoma detection rate is a key colonoscopy quality metric, benchmarks for serrated polyp removal, specifically the sessile serrated lesion detection rate (SSLDR), are currently lacking.

**Objective:**

To evaluate the physician SSLDR and the risk of postcolonoscopy colorectal cancer (PCCRC) and related mortality.

**Design, Setting, and Participants:**

This retrospective cohort study was conducted between January 1, 2000, and December 31, 2021, within a large integrated health care system in Northern California and included colonoscopies performed for patients aged 18 years or older, without a diagnosis of colorectal cancer within 6 months of colonoscopy, history of colorectal cancer, or history of inflammatory bowel disease by 50 gastroenterologists. Statistical analysis was performed from January 1, 2000, to December 31, 2021.

**Exposure:**

The primary exposure was the SSLDR, defined as the proportion of screening colonoscopies with at least 1 histologically confirmed sessile serrated adenoma or traditional serrated adenoma, categorized into quartiles.

**Main Outcomes and Measures:**

The primary outcome was PCCRC diagnosed more than 6 months after negative colonoscopy results. Secondary outcomes included proximal or distal PCCRC, advanced stage colorectal cancer, all-cause mortality, and colorectal cancer–related mortality. Outcomes were ascertained through linked state and institutional cancer registries and vital records.

**Results:**

Among 328 416 colonoscopies performed for 226 695 unique patients (mean [SD] age, 58.6 [10.7] years; 51.7% women), 562 PCCRC cases were identified over 2 038 816 person-years of follow-up. The absolute PCCRC incidence rate decreased with higher SSLDR quartiles, from 3.9 cases per 10 000 person-years in the lowest quartile to 2.4 cases per 10 000 person-years in the highest quartile. Compared with patients in the lowest SSLDR quartile, those in the highest SSLDR quartile had a significantly lower risk of PCCRC (multivariate hazard ratio, 0.69; 95% CI, 0.50-0.94; *P* = .01 for trend), primarily associated with lower risk of proximal PCCRC. A higher SSLDR was associated with lower all-cause mortality (second quartile, 57.7 deaths per 10 000 person-years; third quartile, 58.0 deaths per 10 000 person-years) and colorectal cancer–related mortality (second quartile, 0.2 deaths per 10 000 person-years; third quartile, 0.2 deaths per 10 000 person-years) in the second and third SSLDR quartiles, but the association was not significant in the highest quartile compared with the lowest quartile.

**Conclusions and Relevance:**

In this large community-based cohort study, a higher physician SSLDR was associated with a significantly lower risk of PCCRC in a dose-dependent manner. These findings support using the SSLDR as a crucial quality metric for colonoscopy.

## Key Points

**Question:**

Is the sessile serrated lesion detection rate (SSLDR) associated with lower risk of postcolonoscopy colorectal cancer (PCCRC)?

**Findings:**

In this cohort study comprising 328 416 colonoscopies performed for 226 695 unique patients, the SSLDR was significantly associated with lower risk of PCCRC. The highest SSLDR quartile had a statistically significantly lower multivariate hazard ratio of 0.69 compared with the lowest quartile.

**Meaning:**

This study’s findings suggest that the SSLDR should be used as a quality metric for colonoscopy.

## Teaser

This cohort study evaluates whether the sessile serrated lesion detection rate is associated with risk of postcolonoscopy colorectal cancer and related mortality.

## Introduction

Sessile serrated lesions (SSLs) are colonic polyps characterized by a “saw-toothed” appearance on crypt epithelium. Originally considered benign, SSLs are now recognized as distinct precursor lesions from the traditional adenoma-to-carcinoma sequence leading to colorectal cancer (CRC). Approximately 20% of cases of sporadic CRC arise from SSLs, which are associated with epigenetic methylation and *BRAF* variations.^1,2^ They often occur in the proximal colon and have subtle endoscopic features, making them challenging to detect.

Several studies have shown that SSLs are associated with increased CRC risk.^3,4,5,6,7,8^A randomized clinical trial found that SSLs were associated with a 2.5-fold increased CRC risk and remained an independent risk factor after adjusting for size and concomitant adenomas.^9^ A community-based case-control study similarly reported that SSLs were associated with a 2.9-fold increased CRC risk.^5^ However, few longitudinal studies have investigated the association of SSL removal with long-term CRC incidence and mortality. One Dutch study found that for each 1% increase in the proximal SSL detection rate, there was a 7% decrease in the incidence of postcolonoscopy CRC (PCCRC).^10^

Currently, US multisociety guidelines have recommended using quality benchmarks such as the adenoma detection rate (ADR) to assess the quality of colonoscopies for conventional adenomas.^11^ For SSLs, the guidelines suggest a benchmark SSL detection rate (SSLDR) of 6% or more; however, this recommendation is based on low-quality evidence.^12^ The primary goal of this study is to examine the association of the SSLDR with PCCRC risk and mortality within a large community-based health care system.

## Methods

### Study Setting

This was a retrospective cohort study using data from Sutter Health–Palo Alto Medical Foundation (PAMF), an integrated health care system in Northern California. PAMF serves more than 1 million patients annually across 50 clinical sites and 8 ambulatory surgical centers in 4 Bay Area counties. Almost all patients have insurance coverage (16% health maintenance organization, 61% commercial preferred provider organization or fee-for-service, 20% Medicare or Medicaid, and 3% other). The study and waiver of informed consent were approved by the Sutter Health and California Committee for the Protection of Human Subjects institutional review board; the waiver of consent was granted due to the large number of patients involved in the study and the minimal risks posed to the patients. This study followed the Strengthening the Reporting of Observational Studies in Epidemiology (STROBE) reporting guideline for cohort studies.

### Study Sample

**Figure 1.**
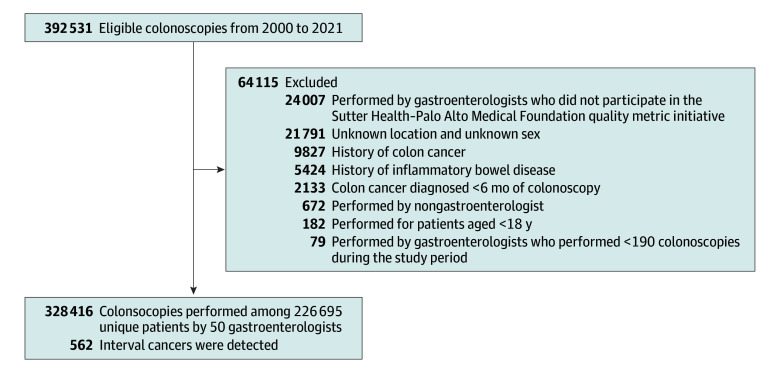
Schematic Flow Diagram

We identified all colonoscopies performed between January 1, 2000, and December 31, 2021, using *Current Procedural Terminology* codes. Indications for colonoscopy were classified using *International Classification of Diseases, Ninth Revision*, and *International Statistical Classification of Diseases and Related Health Problems, Tenth Revision*, codes as follows: screening (asymptomatic individuals without a history of polyps or CRC), surveillance (performed for patients with a history of colon polyps), and diagnostic (performed for symptoms or a positive fecal occult test result). Of 392 531 colonoscopies performed during the study period, we excluded colonoscopies performed by nongastroenterologists (n = 672), performed for patients younger than 18 years (n = 182), performed by gastroenterologists not participating in PAMF’s quality metric initiative (n = 24 007) or with fewer than 190 total examinations in the study period (n = 79), and colonoscopies with unknown location or unknown patient sex (n = 21 791). We further excluded colonoscopies for patients with a CRC diagnosis less than 6 months after the procedure (n = 2133), with a history of CRC (n = 9827), or with a history of inflammatory bowel disease (n = 5424). The final cohort included 328 416 colonoscopies from 226 695 unique patients performed by 50 gastroenterologists (Figure 1). We collected patient-related characteristics including age, sex, body mass index (BMI [calculated as weight in kilograms divided by height in meters squared]), self-reported race and ethnicity from the electronic health system (African American, Asian, White, other [American Indian or Alaska Native, Native Hawaiian or Other Pacific Islander, multiracial, other race and ethnicity, or race and ethnicity not otherwise specified]), Charlson Comorbidity Index (CCI), smoking history (never, current, former, or unknown), removal of conventional adenoma (yes or no), and indication for colonoscopy (screening or surveillance vs diagnostic). Race and ethnicity are collected as part of routine care in our health care system, which is for available for analytics. We defined conventional adenomas as those being detected by colonoscopies in which 1 or more tubular, tubulovillous, or villous adenoma was removed. PAMF is an open health care system, and pathology reports from outside facilities were not consistently available electronically; these records were therefore classified as unknown.

### Sessile Serrated Lesion Detection Rate

Beginning in 2015, the organization implemented a system-wide initiative to improve the quality metrics of colonoscopies. Physicians manually reported polyp pathology for each screening or surveillance colonoscopy, including the number and type of polyps (tubular, tubulovillous, villous, SSL, or traditional serrated adenoma). SSLs were defined as sessile serrated adenomas or traditional serrated adenomas. We calculated each physician’s SSLDR as the proportion of colonoscopies with SSLs among screening colonoscopies (2015-2021) and categorized the SSLDR into quartiles. Subsequently, each colonoscopy during the study period was assigned to the performing physician’s SSLDR. The SSLDR was collected as part of routine quality indicators reviewed quarterly at ambulatory surgical centers. The adherence rate of polyp reporting was close to 100% across all gastroenterologists (eMethods in Supplement 1).

### CRC, Mortality, and Stage

Data on incident CRC, including location and stage, were obtained from Sutter and California Cancer Registries. We classified CRC as proximal (cecum, ascending, hepatic flexure, or transverse [codes C180-184]) or distal (splenic flexure, descending, or sigmoid colon or rectum [codes C185-C187, C199, and C209]) according to Surveillance, Epidemiology, and End Results (SEER) *International Classification of Diseases for Oncology, 3rd Edition*, site codes.^13,14^ Cancer stage was categorized as early (stage I or II) or advanced (stage III or IV) per SEER–American Joint Committee on Cancer 8th edition staging schema.^15^ Mortality data were obtained from the state cancer registry and Sutter death records. Each CRC diagnosed was matched with the corresponding individual’s colonoscopy. We defined PCCRC as CRC diagnosed 6 months or more after a negative colonoscopy result.^10,12,16^

### Statistical Analysis

Statistical analysis was performed from January 1, 2000, to December 31, 2021. We calculated descriptive statistics overall and by SSLDR quartiles (first quartile: 0.3%-6.4%; second quartile: 6.5%-10.3%; third quartile: 10.4%-14.6%; fourth quartile: 14.7%-24.2%). Age-adjusted hazard ratios (HRs) and 95% CIs for PCCRC were estimated using Cox proportional hazards regression with SSLDR quartiles as the independent variable. For PCCRC risk, person-time was calculated from the date of colonoscopy until earliest CRC diagnosis, death from any cause, or end of study (December 31, 2021).^10^ We calculated HRs of PCCRC, proximal PCCRC, distal PCCRC, and advanced stage CRC separately using competing risk models with the Fine and Gray method.^17^ In addition, we calculated the HRs for all-cause and CRC-specific mortality by SSLDR quartiles. For mortality analyses, person-time was calculated from the date of colonoscopy until death from any cause (all-cause mortality) or end of follow-up, whichever came first.^18^

In the multivariate analysis, we adjusted for age (18-50, 51-75, or >75 years), sex (female or male), BMI (<25, 25-30, or >30), race and ethnicity (African American, Asian, White, or other), smoking history (current, former, never, or unknown), CCI (0 or ≥1), indication of colonoscopy (diagnostic, screening, or surveillance), and removal of conventional adenoma (no, yes, or unknown). We performed a linear trend test of the SSLDR on PCCRC risk and mortality. We used a robust sandwich variance estimate to account for within-individual and physician clustering.^13,19,20^ We tested the proportional hazard assumption using the Kolmogorov-type supremum test, which did not reject the assumption. To ensure the robustness of the results, we performed sensitivity analyses using different censoring times, including at next colonoscopy.^19,20,21^ We also performed a stratified analysis to evaluate the association of ADR with SSLDR and PCCRC risk. To address potential socioeconomic associations with PCCRC risk, we performed a secondary analysis adjusting for median household income based on zip code.

Kaplan-Meier curves were constructed for cumulative PCCRC incidence for each SSLDR quartile, showing risk at various time points after colonoscopy. We calculated the unadjusted PCCRC risk per 10 000 person-years. Analyses were conducted using SAS, version 9.4 (SAS Institute Inc). All *P* values were from 2-sided tests and results were deemed statistically significant at *P* < .05.

## Results

### Baseline Characteristics

Overall baseline characteristics of the study population are presented in Table 1. We analyzed 328 416 colonoscopies performed for 226 695 unique patients (mean [SD] patient age, 58.6 [10.7] years; 51.7% women and 48.3% men; 1.7% African American, 20.7% Asian, 61.7% White, and 15.9% other race or ethnicity; mean [SD] BMI, 27.3 [5.7]) with 562 PCCRC cases identified over 2 038 816 person-years of follow-up. The median time between colonoscopy and PCCRC was 5.5 years (IQR, 3.3-8.5 years). Never smokers represented 63.8% of patients, followed by former smokers (23.8%), current smokers (3.7%), and unknown smoking status (8.7%). The mean (SD) CCI was 1.8 (2.3). Most procedures (71.6%) were screening or surveillance, while 28.4% were diagnostic. At least 1 conventional adenoma was removed in 31.4% of procedures, while 58.7% had no adenoma and 9.9% had unknown adenoma status. From 2015 to 2021, among endoscopists, the overall median SSLDR was 10.3% (IQR, 6.5%-14.4%), and the median ADR was 42.4% (IQR, 38.3%-51.5%) (eTable 1 in Supplement 1).

**Table 1.** Baseline Characteristics

Characteristic	Overall (N = 328 416)^a^	Sessile serrated lesion detection rate			
		First quartile, 0.3%-6.4% (n = 45 869)	Second quartile, 6.5%-10.3% (n = 119 056)	Third quartile, 10.4%-14.6% (n = 79 240)	Fourth quartile, 14.7%-24.2% (n = 84 251)
Age, mean (SD), y	58.6 (10.7)	59.8 (10.7)	58.1 (10.7)	58.5 (10.5)	58.5 (10.9)
Age group, No. (%)					
18-50 y	60 285 (18.4)	7454 (16.3)	23 554 (19.8)	14 382 (18.1)	14 895 (17.7)
51-75 y	248 368 (75.6)	35 183 (76.7)	88 464 (74.3)	60 549 (76.4)	64 172 (76.2)
>75 y	19 763 (6.0)	3232 (7.0)	7038 (5.6)	4309 (8.6)	5184 (8.6)
Sex, No. (%)					
Male	158 647 (48.3)	23 616 (51.5)	62 925 (52.9)	32 397 (40.9)	39 709 (47.1)
Female	169 769 (51.7)	22 253 (48.5)	56 131 (47.1)	46 843 (59.1)	44 542 (52.9)
BMI, mean (SD)	27.3 (5.7)	27.7 (5.4)	27.1 (5.3)	27.2 (6.6)	27.4 (5.5)
BMI category, No. (%)					
<25	118 458 (36.1)	14 472 (31.6)	43 984 (36.9)	30 304 (38.2)	29 698 (35.2)
25-30	118 464 (36.1)	16 949 (37.0)	43 499 (36.5)	27 614 (34.8)	30 402 (36.1)
>30	79 313 (24.2)	11 894 (25.9)	27 323 (22.9)	18 838 (23.8)	21 258 (25.2)
Unknown	12 181 (3.7)	2554 (5.6)	4250 (3.6)	2484 (3.1)	2893 (3.4)
Race and ethnicity, No. (%)					
African American	5536 (1.7)	631 (1.4)	1966 (1.7)	1616 (2.0)	1323 (1.6)
Asian	68 108 (20.7)	7415 (16.2)	29 670 (24.9)	19 059 (24.1)	11 964 (14.2)
White	202 682 (61.7)	30 725 (67.0)	68 714 (57.7)	45 681 (57.6)	57 562 (68.3)
Other^b^	52 090 (15.9)	15 240 (15.3)	6214 (14.4)	4641 (15.6)	25 995 (16.7)
Smoking status, No. (%)					
Never smoker	209 400 (63.8)	24 559 (53.5)	77 169 (64.8)	52 429 (66.2)	55 243 (65.6)
Current smoker	12 107 (3.7)	1752 (3.8)	3840 (3.2)	2792 (3.5)	3723 (4.4)
Former smoker	78 278 (23.8)	10 895 (23.8)	24 943 (21.0)	19 132 (24.1)	23 308 (27.7)
Unknown	28 631 (8.7)	8663 (18.9)	13 104 (11.0)	4887 (6.2)	1977 (2.3)
Charlson Comorbidity Index, mean (SD)	1.8 (2.3)	2.1 (2.6)	1.8 (2.2)	1.8 (2.3)	1.8 (2.2)
Charlson Comorbidity Index, No. (%)					
0	118 179 (36.0)	15 814 (34.5)	43 321 (36.4)	27 555 (34.8)	31 489 (37.4)
≥1	210 370 (64.1)	30 055 (65.5)	75 735 (63.6)	51 685 (65.2)	52 762 (62.6)
Colonoscopy indication, No. (%)					
Screening or surveillance	235 069 (71.6)	28 283 (61.7)	89 537 (75.2)	58 594 (73.9)	58 655 (69.6)
Diagnostic	93 347 (28.4)	17 586 (38.3)	29 519 (24.8)	20 646 (26.1)	25 596 (30.4)
Conventional adenoma removed, No. (%)^c^					
Yes	103 119 (31.4)	8187 (17.8)	40 568 (34.1)	23 587 (29.8)	30 777 (36.5)
No	192 709 (58.7)	39 487 (72.0)	73 183 (61.7)	46 215 (58.3)	42 811 (50.8)
Unknown	32 588 (9.9)	7096 (12.9)	4523 (3.8)	8307 (10.5)	10 346 (12.3)

Abbreviation: BMI, body mass index (calculated as weight in kilograms divided by height in meters squared).

^a^ Includes 328 416 colonoscopies among 226 695 unique patients.

^b^ Other includes American Indian or Alaska Native, Native Hawaiian or Other Pacific Islander, multiraceial, other race and ethnicity, or race and ethnicity not otherwise specified.

^c^ Any tubular adenoma, tubulovillous, and/or villous adenoma removed.

Compared with the lowest SSLDR quartile, colonoscopies in the highest quartile were performed for patients who were slightly younger, were more likely to be female, were current or former smokers, underwent a screening or surveillance colonoscopy, had lower CCI scores, and had more conventional adenomas removed. In contrast, race and ethnicity and BMI distributions appeared similar across quartiles.

### SSLDR and Risk of PCCRC

**Figure 2.**
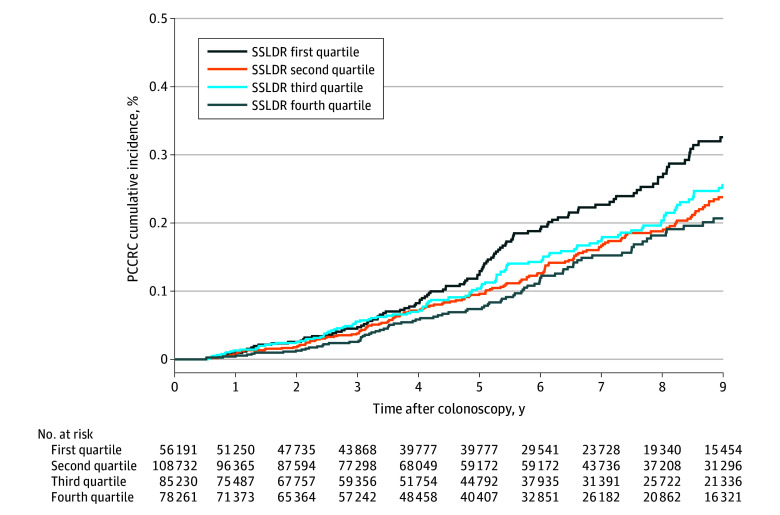
Cumulative Incidence of Postcolonoscopy Colorectal Cancer (PCCRC) According to Sessile Serrated Lesion Detection Rate (SSLDR) by Quartile

The absolute PCCRC incidence rate was inversely associated with a higher SSLDR (first quartile, 3.9 PCCRC per 10 000 person-years; second quartile, 2.5 PCCRC per 10 000 person-years; third quartile, 2.6 PCCRC per 10 000 person-years; and fourth quartile, 2.4 PCCRC per 10 000 person-years), as illustrated in the Kaplan-Meier curve (Figure 2). Patients in the highest SSLDR quartile had a significantly lower risk of PCCRC (age-adjusted HR, 0.67; 95% CI, 0.49-0.91) compared with those in the lowest SSLDR quartile (Table 2). After adjusting for covariates, the association remained unchanged (multivariate HR [MHR], 0.69; 95% CI, 0.50-0.94). Compared with patients in the lowest SSLDR quartile, those in the second quartile had an MHR of 0.73 (95% CI, 0.55-0.96) and those in the third quartile had an MHR of 0.74 (95% CI, 0.55-0.99). PCCRC risk showed a dose-dependent inverse association with SSLDR, with patients in the highest SSLDR quartile having the lowest risk of PCCRC (continuous HR, 0.97; 95% CI, 0.95-0.99; *P* = .01 for trend).

**Table 2.** Risk of PCCRC According to SSLDR Quartiles

Characteristic	First quartile SSLDR	Second quartile SSLDR	Third quartile SSLDR	Fourth quartile SSLDR	*P* value for trend (linear)
**All PCCRC^a^**					
No. of cases/person-year	146/375 360	174/692 316	134/517 582	108/453 559	NA
Incidence rate, No. of cases/10 000 person-years	3.9	2.5	2.6	2.4	NA
Age-adjusted HR (95% CI)	1.0 [Reference]	0.68 (0.51-0.90)	0.70 (0.52-0.94)	0.67 (0.49-0.91)	.02
Multivariate HR (95% CI)^b^	1.0 [Reference]	0.73 (0.55-0.96)	0.74 (0.55-0.99)	0.69 (0.50-0.94)	.01
**Proximal PCCRC^c^**					
No. of cases/person-year	77/375 360	68/692 316	57/517 582	41/453 559	NA
Incidence rate, No. of cases/10 000 person-years	2.1	1.0	1.1	0.9	NA
Age-adjusted HR (95% CI)	1.0 [Reference]	0.51 (0.32-0.82)	0.57 (0.36-0.89)	0.48 (0.30-0.77)	.01
Multivariate HR (95% CI)	1.0 [Reference]	0.59 (0.37-0.93)	0.60 (0.38-0.94)	0.51 (0.32-0.81)	.007
**Distal PCCRC^d^**					
No. of cases/person-year	61/375 360	101/692 316	71/517 582	61/453 559	NA
Incidence rate, No. of cases/10 000 person-years	1.6	1.5	1.4	1.3	NA
Age-adjusted HR (95% CI)	1.0 [Reference]	0.93 (0.64-1.35)	0.89 (0.59-1.34)	0.90 (0.59-1.38)	.43
Multivariate HR (95% CI)	1.0 [Reference]	0.93 (0.64-1.37)	0.95 (0.63-1.42)	0.93 (0.60-1.44)	.56
**Advanced stage PCCRC^e^**					
No. of deaths/person-year	41/375 360	46/692 316	42/517 582	35/453 559	NA
Incidence rate, No. of cases/10 000 person-years	1.1	0.7	0.8	0.8	NA
Age-adjusted HR (95% CI)	1.0 [Reference]	0.64 (0.39-1.05)	0.79 (0.48-1.32)	0.79 (0.45-1.39)	.63
Multivariate HR (95% CI)	1.0 [Reference]	0.70 (0.42-1.15)	0.82 (0.48-1.40)	0.81 (0.46-1.44)	.53

Abbreviations: HR, hazard ratio; NA, not applicable; PCCRC, postcolonoscopy colorectal cancer; SSLDR, sessile serrated lesion detection rate.

^a^ Includes 25 cases of colorectal cancer in an unknown location.

^b^ Multivariate analysis included age, sex, body mass index, race and ethnicity (African American, Asian, White, other [American Indian or Alaska Native, Native Hawaiian or Other Pacific Islander, multiracial, other race and ethnicity, or race and ethnicity not otherwise specified]), smoking history, Charlson Comorbidity Index, indication of colonoscopy, and removal of conventional adenoma.

^c^ Proximal PCCRC includes cancer in the appendix, cecum, ascending colon, hepatic flexure, and transverse colon.

^d^ Distal PCCRC includes cancer in the splenic flexure, descending colon, sigmoid colon, rectosigmoid colon, and rectum.

^e^ Advanced stage PCCRC includes stage 3 or 4 colorectal cancer.

As a sensitivity analysis, we repeated the analysis adding censoring at the next colonoscopy. We found similar results, with patients in the highest SSLDR quartile having a significantly lower PCCRC risk (MHR, 0.74; 95% CI, 0.55-0.99; *P* = .03 for trend) compared with those in the lowest quartile. Although we adjusted for the presence of adenoma in our multivariate analysis, we further explored the associations of ADR with SSLDR and PCCRC risk. Stratified analysis by ADR (>35% and ≤35%) showed no significant differences in the association of SSLDR with PCCRC risk by ADR (>35%: MHR, 0.99; 95% CI, 0.96-1.02; ≤35%: MHR, 0.97; 95% CI, 0.86-1.09; *P* = .80 for interaction). This finding suggests that the association of SSLDR and PCCRC risk is independent of ADR.

To assess the association of colonoscopy indication with SSLDR, we performed a stratified analysis comparing screening and surveillance vs diagnostic colonoscopies. Among screening and surveillance colonoscopies, the SSLDR was not associated with lower PCCRC risk. In contrast, among diagnostic colonoscopies, the SSLDR was associated with lower PCCRC risk (highest SSLDR quartile MHR, 0.53; 95% CI 0.35-0.81) (eTables 2-5 in Supplement 1).

### SSLDR and Risk of Proximal, Distal, and Advanced Stage PCCRC

To assess the association between SSLDR and PCCRC location, we categorized PCCRC cases as proximal or distal (Table 2). There were 243 proximal, 294 distal, and 25 unknown PCCRC cases. Similar to overall PCCRC, the absolute incidence rate of proximal PCCRC was inversely associated with increasing SSLDR (first quartile, 2.1 proximal PCCRC per 10 000 person-years; second quartile, 1.0 proximal PCCRC per 10 000 person-years; third quartile, 1.1 proximal PCCRC per 10 000 person-years; fourth quartile, 0.9 proximal PCCRC per 10 000 person-years; *P* = .01 for trend). Compared with patients in the lowest SSLDR quartile, those in the highest quartile had a significantly lower risk of proximal PCCRC (MHR, 0.51; 95% CI, 0.32-0.81), with similar inverse associations in the second and third quartiles (second quartile: MHR, 0.59; 95% CI, 0.37-0.93; third quartile: MHR, 0.60; 95% CI, 0.38-0.94). In contrast, no significant association was found between SSLDR and distal PCCRC risk (age-adjusted HR, 0.90; 95% CI, 0.59-1.38; MHR, 0.93; 95% CI, 0.60-1.44) comparing the highest with the lowest SSLDR quartiles.

We also evaluated the association of SSLDR with advanced-stage PCCRC. Although higher SSLDR quartiles were associated with a lower risk of PCCRC compared with the lowest quartile, these associations were not statistically significant (Table 2).

### SSLDR and Risk of PCCRC-Related Mortality

We examined the association of SSLDR with both all-cause and CRC-related mortality (Table 3). There were 12 465 all-cause deaths and 64 CRC-related deaths during the study period. A higher SSLDR was associated with a lower absolute incidence of all-cause mortality (first quartile, 74.4 deaths per 10 000 person-years; second quartile, 57.7 deaths per 10 000 person-years; third quartile, 58.0 deaths per 10 000 person-years; fourth quartile, 58.9 deaths per 10 000 person-years). Compared with the lowest SSLDR quartile, all-cause mortality risk was significantly lower in the second, third, and fourth SSLDR quartiles (second quartile: age-adjusted HR, 0.83; 95% CI, 0.78-0.88; third quartile: age-adjusted HR, 0.84; 95% CI, 0.79-0.89; fourth quartile: age-adjusted HR, 0.91; 95% CI, 0.85-0.97). However, after adjusting for covariates, the association for the highest SSLDR quartile was no longer statistically significant (MHR, 0.96; 95% CI, 0.90-1.02). We did not observe a dose-dependent association between SSLDR and all-cause mortality (*P* = .33 for trend)

**Table 3.** Risk of Mortality According to SSLDR Quartiles

Characteristic	First quartile SSLDR	Second quartile SSLDR	Third quartile SSLDR	Fourth quartile SSLDR	*P* value for trend (linear)
**All-cause mortality**					
No. of deaths/person-year	2795/3 754 885	3996/693 068	3003/518 147	2671/453 854	NA
Incidence rate, No. of cases/10 000 person-years	74.4	57.7	58.0	58.9	NA
Age-adjusted HR (95% CI)	1.0 [Reference]	0.83 (0.78-0.88)	0.84 (0.79-0.89)	0.91 (0.85-0.97)	.88
Multivariate HR (95% CI)^a^	1.0 [Reference]	0.94 (0.89-0.99)	0.88 (0.83-0.94)	0.96 (0.90-1.02)	.33
**PCCRC-related mortality**					
No. of deaths/person-year	25/3 754 885	15/693 068	11/518 147	13/453 854	NA
Incidence rate, No. of cases/10 000 person-years	0.7	0.2	0.2	03	NA
Age-adjusted HR (95% CI)	1.0 [Reference]	0.35 (0.17-0.71)	0.35 (0.17-0.72)	0.59 (0.25-1.12)	.20
Multivariate HR (95% CI)	1.0 [Reference]	0.42 (0.21-0.86)	0.36 (0.18-0.74)	0.59 (0.28-1.25)	.19

Abbreviations: HR, hazard ratio; NA, not applicable; PCCRC, postcolonoscopy colorectal cancer; SSLDR, sessile serrated lesion detection rate.

^a^ Multivariate analysis included age, sex, body mass index, race and ethnicity (African American, Asian, White, other [American Indian or Alaska Native, Native Hawaiian or Other Pacific Islander, multiracial, other race and ethnicity, or race and ethnicity not otherwise specified]), smoking history, Charlson Comorbidity Index, indication of colonoscopy, and removal of conventional adenoma.

Similarly, for CRC-related mortality, higher SSLDR was associated with lower CRC-related death rates (first quartile, 0.7 CRC-related deaths per 10 000 person-years; second quartile, 0.2 CRC-related deaths per 10 000 person-years; third quartile, 0.2 CRC-related deaths per 10 000 person-years; fourth quartile, 0.3 CRC-related deaths per 10 000 person-years) (Table 3). Compared with the lowest SSLDR quartile, the second and third quartiles were significantly associated with lower risk of CRC-related mortality (second quartile: MHR, 0.42; 95% CI, 0.21-0.86; third quartile: MHR, 0.36; 95% CI, 0.18-0.74). However, there was no association of the highest SSLDR quartile with risk of death from PCCRC (MHR, 0.59; 95% CI, 0.28-1.25). The lower associated risk of CRC-related death did not appear to be dose dependent (*P* = .19 for trend).

To examine the lack of association between highest SSLDR and mortality, we adjusted for zip code–level household income. Results remained consistent; the highest SSLDR quartile showed no significant association with all-cause mortality (MHR, 0.97; 95% CI, 0.91-1.04) or PCCRC-related mortality (MHR, 0.59; 95% CI, 0.28-1.26) (eTables 6 and 7 in Supplement 1).

## Discussion

In this large community-based cohort study, we investigated the association between SSLDR and PCCRC. We found that a higher SSLDR was associated with a significantly lower PCCRC risk, showing a dose-dependent inverse association.

Although ample evidence links SSLs with increased risk of CRC,^4,5,6,7,9,22^ few longitudinal studies have specifically investigated the association of SSLDR with long-term CRC rates. Prior research suggests that a higher SSLDR may correlate with lower PCCRC risk, but evidence remains limited.^3,21,22^ Our findings extend prior work by evaluating SSLDR in a large US community-based setting and demonstrating its link with reduced PCCRC risk, particularly for proximal cancers, which is consistent with the predominantly proximal location of SSLs.^1,23^

Recent guidelines have proposed a minimum SSLDR threshold of 6% as a colonoscopy quality indicator.^24^ However, this recommendation is based on weak and limited evidence. Reliability concerns remain due to interobserver variation in the diagnosis of SSLs among pathologists and the low prevalence of SSLs, which can affect SSLDR accuracy. Our study adds new insight to the proper SSLDR threshold. Although we observed a significant linear trend between SSLDR quartiles and PCCRC risk, the dose-response association remains uncertain. Most of the PCCRC risk reduction occurred from the second quartile, with a smaller incremental reduction at higher quartiles, consistent with the literature.^3^ Our second quartile was measured starting at 6.4%, supporting the use of a 6% SSLDR benchmark. However, other studies have suggested a true gradient between SSLDR and PCCRC risk.^10^ Given the increasing detection of SSLs over time,^25,26^ further research is needed to determine whether a fixed or time-adjusted SSLDR threshold is most appropriate.

A higher SSLDR was also associated with a lower risk of both all-cause and CRC-related mortality, although the association was not significant in the highest SSLDR quartile. Few studies have examined the SSLDR and mortality. An Austrian study found that a higher SSLDR was associated with lower PCCRC-related mortality, primarily associated with the detection of proximal SSLs.^18^ These findings align with ours and are biologically plausible given the proximal location of SSLs, further supporting that a higher SSLDR may be associated with reduced CRC-related mortality. Although the Austrian study did not specifically examine all-cause mortality, our findings suggest that a higher SSLDR may also be associated with reduced all-cause mortality. However, this should be interpreted cautiously as significance was observed only in the second and third quartiles, not in the highest quartile, suggesting that other unmeasured factors may be influencing this finding and should be validated in future studies.

### Strengths and Limitations

Several strengths of this study are worth noting. We used data from a large, diverse, community-based population, enhancing the generalizability of our findings. With over 20 years of follow-up, we were able to account for cases of PCCRC that occurred several years after negative colonoscopy results. Our analysis used data linked to the Sutter and California Cancer registry and the state vital statistics to capture CRC cases and mortality, leading to more robust ascertainment of outcomes. Moreover, we used physician-reviewed and physician-interpreted pathology to calculate the SSLDR, enhancing the accuracy of SSL identification over approaches relying solely on automated coding or natural language processing, which might be prone to misclassification.

Several key limitations should also be considered. First, the changing nomenclature of SSLs may have affected the identification and calculation of the SSLDR. Prior to 2010, most SSLs were classified as benign hyperplastic polyps. A 2019 review article noted that the World Health Organization (WHO) 2010 redefinition of SSLs resulted in a 19% reclassification rate of hyperplastic polyps into SSLs, whereas using the WHO definition from 2019 resulted in only a 7% rediagnosis rate.^1^ Our SSLDR calculation began in 2015, well after the 2010 WHO reclassification, mitigating widespread misclassification of SSLs during the study period. Moreover, physician-reviewed pathology reports were also used to resolve interpretation differences. Second, other confounding risk factors, such as alcohol use, physical activity, and dietary factors, have been reported elsewhere to be associated with increased risk of SSLs but could not be reliably accounted for here. Nevertheless, these would minimally affect the SSLDR, as it is an endoscopist-specific quality metric. Third, the ADR is an established quality metric associated with lower PCCRC risk.^13,20,24,27^ Some studies found that endoscopists with a high ADR also tend to have a higher SSLDR, as conventional adenomas are often frequently seen in patients with SSLs,^28,29^ which raises the possibility that our findings could reflect the concurrent effects of ADR rather than SSLDR alone. However, more recent studies showed that the SSLDR may be independently associated with PCCRC.^10,18,21^ To address this, we adjusted for conventional adenomas and conducted stratified analyses by the ADR, which showed that the SSLDR remained associated with lower PCCRC risk independent of the ADR. Fourth, we could not reliably classify index from surveillance colonoscopies due to the open nature of the PAMF network, which may have led to misclassification and could obscure associations between SSL detection at the index procedure and CRC outcomes.

## Conclusions

In this cohort study of a large community-based health care system, we found evidence that a higher SSLDR was significantly associated with a lower PCCRC risk, associated predominantly with a reduction in proximal CRCs. These findings underscore the importance of the effective detection and removal of SSLs and support the use of the SSLDR as a crucial quality metric for colonoscopy.

## Notes

Supplement 1. **eMethods.** Quality Metric Initiative, Natural Language Processing and Adherence
eReference.
**eTable 1.** SSLDR and ADR at Provider Level **eTable 2.** Risk of PCCRC According to SSLDR Quartiles Among Screening and Surveillance Colonoscopies (n=235,069) **eTable 3.** Risk of Mortality According to SSLDR Quartiles Among Screening and Surveillance Colonoscopies (n=235,069) **eTable 4.** Risk of PCCRC According to SSLDR Quartiles Among Diagnostic Colonoscopy (n=93,347) **eTable 5.** Risk of Mortality According to SSLDR Quartiles Among Diagnostic Colonoscopy (n=93,347) **eTable 6.** Baseline Characteristics With Income **eTable 7.** Risk of Mortality According to SSLDR Quartiles Adjusting for Income

Supplement 2.
Data Sharing Statement

## References

[1] Crockett SD, Nagtegaal ID. Terminology, molecular features, epidemiology, and management of serrated colorectal neoplasia. Gastroenterology. 2019;157(4):949-966. doi:10.1053/j.gastro.2019.06.041 31323292

[2] Crockett SD, Snover DC, Ahnen DJ, Baron JA. Sessile serrated adenomas: an evidence-based guide to management. Clin Gastroenterol Hepatol. 2015;13(1):11-26. doi:10.1016/j.cgh.2013.10.035 24216467

[3] Anderson JC, Rex DK, Mackenzie TA, Hisey W, Robinson CM, Butterly LF. Higher serrated polyp detection rates are associated with lower risk of postcolonoscopy colorectal cancer: data from the New Hampshire Colonoscopy Registry. Am J Gastroenterol. 2023;118(11):1927-1930. doi:10.14309/ajg.0000000000002403 37417792 PMC10841069

[4] He X, Hang D, Wu K, . Long-term risk of colorectal cancer after removal of conventional adenomas and serrated polyps. Gastroenterology. 2020;158(4):852-861. doi:10.1053/j.gastro.2019.06.039 31302144 PMC6954345

[5] Li D, Doherty AR, Raju M, . Risk stratification for colorectal cancer in individuals with subtypes of serrated polyps. Gut. Published online August 11, 2021. doi:10.1136/gutjnl-2021-32 34380653

[6] Li D, Liu L, Fevrier HB, . Increased risk of colorectal cancer in individuals with a history of serrated polyps. Gastroenterology. 2020;159(2):502-511. doi:10.1053/j.gastro.2020.04.004 32277950

[7] Polychronidis G, He MM, Vithayathil M, Knudsen MD, Wang K, Song M. Risk of colorectal neoplasia after removal of conventional adenomas and serrated polyps: a comprehensive evaluation of risk factors and surveillance use. Gut. 2024;73(10):1675-1683. doi:10.1136/gutjnl-2023-331729 38839270

[8] Song M, Emilsson L, Bozorg SR, . Risk of colorectal cancer incidence and mortality after polypectomy: a Swedish record-linkage study. Lancet Gastroenterol Hepatol. 2020;5(6):537-547. doi:10.1016/S2468-1253(20)30009-1 32192628 PMC7234902

[9] Holme Ø, Bretthauer M, Eide TJ, . Long-term risk of colorectal cancer in individuals with serrated polyps. Gut. 2015;64(6):929-936. doi:10.1136/gutjnl-2014-307793 25399542

[10] van Toledo DEFWM, IJspeert JEG, Bossuyt PMM, . Serrated polyp detection and risk of interval post-colonoscopy colorectal cancer: a population-based study. Lancet Gastroenterol Hepatol. 2022;7(8):747-754. doi:10.1016/S2468-1253(22)00090-5 35550250

[11] Gupta S, Lieberman D, Anderson JC, . Spotlight: US Multi-Society Task Force on Colorectal Cancer recommendations for follow-up after colonoscopy and polypectomy. Gastroenterology. 2020;158(4):1154. doi:10.1053/j.gastro.2020.02.014 32063515 PMC7687298

[12] Anderson JC, Butterly LF. Assessing risk of index serrated polyps. Clin Gastroenterol Hepatol. 2024;22(5):958-960. doi:10.1016/j.cgh.2023.10.020 37924854 PMC12869790

[13] Corley DA, Jensen CD, Marks AR, . Adenoma detection rate and risk of colorectal cancer and death. N Engl J Med. 2014;370(14):1298-1306. doi:10.1056/NEJMoa1309086 24693890 PMC4036494

[14] ICD-O-3 coding materials. National Cancer Institute; Surveillance, Epidemiology, and End Results Program. Accessed December 29, 2025. https://seer.cancer.gov/icd-o-3/

[15] Amin MB, Edge SB, Greene FL, et al, eds. *AJCC Cancer Staging Manual*. 8th ed. Springer; 2017.

[16] Rutter MD, Beintaris I, Valori R, . World Endoscopy Organization consensus statements on post-colonoscopy and post-imaging colorectal cancer. Gastroenterology. 2018;155(3):909-925. doi:10.1053/j.gastro.2018.05.038 29958856

[17] Fine JP, Gray RJ. A proportional hazards model for the subdistribution of a competing risk. J Am Stat Assoc. 1999;94(446):496-509. doi:10.1080/01621459.1999.10474144

[18] Zessner-Spitzenberg J, Waldmann E, Jiricka L, . Comparison of adenoma detection rate and proximal serrated polyp detection rate and their effect on post-colonoscopy colorectal cancer mortality in screening patients. Endoscopy. 2023;55(5):434-441. doi:10.1055/a-1974-9979 36482285

[19] Wieszczy P, Bugajski M, Januszewicz W, . Comparison of quality measures for detection of neoplasia at screening colonoscopy. Clin Gastroenterol Hepatol. 2023;21(1):200-209. doi:10.1016/j.cgh.2022.03.023 35341951

[20] Schottinger JE, Jensen CD, Ghai NR, . Association of physician adenoma detection rates with postcolonoscopy colorectal cancer. JAMA. 2022;327(21):2114-2122. doi:10.1001/jama.2022.6644 35670788 PMC9175074

[21] Anderson JC, Hisey W, Mackenzie TA, . Clinically significant serrated polyp detection rates and risk for postcolonoscopy colorectal cancer: data from the New Hampshire Colonoscopy Registry. Gastrointest Endosc. 2022;96(2):310-317. doi:10.1016/j.gie.2022.03.001 35276209 PMC9296608

[22] Erichsen R, Baron JA, Hamilton-Dutoit SJ, . Increased risk of colorectal cancer development among patients with serrated polyps. Gastroenterology. 2016;150(4):895-902. doi:10.1053/j.gastro.2015.11.046 26677986

[23] Bailie L, Loughrey MB, Coleman HG. Lifestyle risk factors for serrated colorectal polyps: a systematic review and meta-analysis. Gastroenterology. 2017;152(1):92-104. doi:10.1053/j.gastro.2016.09.003 27639804

[24] Rex DK, Anderson JC, Butterly LF, . Quality indicators for colonoscopy. Gastrointest Endosc. 2024;100(3):352-381. doi:10.1016/j.gie.2024.04.2905 39177519 PMC13192241

[25] Liang SY, Oscarson B, Kenkare P, . Trends in detection of adenoma and sessile serrated lesions over a decade in a community-based healthcare system. Clin Transl Gastroenterol. 2024;15(3):e00683. doi:10.14309/ctg.0000000000000683 38270213 PMC10962881

[26] Shaukat A, Holub J, Greenwald D, Eisen G, Schmitt C. Variation over time and factors associated with detection rates of sessile serrated lesion across the United States: results form a national sample using the GIQuIC Registry. Am J Gastroenterol. 2021;116(1):95-99. doi:10.14309/ajg.0000000000000824 32833735

[27] Kaminski MF, Regula J, Kraszewska E, . Quality indicators for colonoscopy and the risk of interval cancer. N Engl J Med. 2010;362(19):1795-1803. doi:10.1056/NEJMoa0907667 20463339

[28] Zorzi M, Senore C, Da Re F, ; Equipe Working Group. Detection rate and predictive factors of sessile serrated polyps in an organised colorectal cancer screening programme with immunochemical faecal occult blood test: the EQuIPE study (Evaluating Quality Indicators of the Performance of Endoscopy). Gut. 2017;66(7):1233-1240. doi:10.1136/gutjnl-2015-310587 26896459

[29] Ohki D, Tsuji Y, Shinozaki T, . Sessile serrated adenoma detection rate is correlated with adenoma detection rate. World J Gastrointest Oncol. 2018;10(3):82-90. doi:10.4251/wjgo.v10.i3.82 29564038 PMC5852399

