# Supplementary material for: Sessile Serrated Lesion Detection Rate and Colorectal Cancer Risk and Mortality
Source: JAMA Netw Open. 2026 Feb 17;9(2):e2556964. doi: 10.1001/jamanetworkopen.2025.56964 (PMC12914494; doi:10.1001/jamanetworkopen.2025.56964)
Supplement: Supplement 1. — eMethods. Quality Metric Initiative, Natural Language Processing and Adherence eReference. eTable 1. SSLDR and ADR at Provider Level eTable 2. Risk of PCCRC According to SSLDR Quartiles Among Screening and Surveillance Colonoscopies (n=235,069) eTable 3. Risk of Mortality According to SSLDR Quartiles Among Screening and Surveillance Colonoscopies (n=235,069) eTable 4. Risk of PCCRC According to SSLDR Quartiles Among Diagnostic Colonoscopy (n=93,347) eTable 5. Risk of Mortality According to SSLDR Quartiles Among Diagnostic Colonoscopy (n=93,347) eTable 6. Baseline Characteristics With Income eTable 7. Risk of Mortality According to SSLDR Quartiles Adjusting for Income [file jamanetwopen-e2556964-s001.pdf]

Supplementary Online Content

Huang ES, Huang Q, Kenkare P, et al. Sessile serrated lesion detection rate and colorectal cancer risk and mortality. *JAMA Netw Open.* 2026;9(2):e2556964. doi:10.1001/jamanetworkopen.2025.56964

eMethods. Quality Metric Initiative, Natural Language Processing and Adherence
eReference.
eTable 1. SSLDR and ADR at Provider Level
eTable 2. Risk of PCCRC According to SSLDR Quartiles Among Screening and Surveillance Colonoscopies (n=235,069)
eTable 3. Risk of Mortality According to SSLDR Quartiles Among Screening and Surveillance Colonoscopies (n=235,069)
eTable 4. Risk of PCCRC According to SSLDR Quartiles Among Diagnostic Colonoscopy (n=93,347)
eTable 5. Risk of Mortality According to SSLDR Quartiles Among Diagnostic Colonoscopy (n=93,347)
eTable 6. Baseline Characteristics With Income
eTable 7. Risk of Mortality According to SSLDR Quartiles Adjusting for Income

This supplementary material has been provided by the authors to give readers additional information about their work.

© 2026 Huang ES et al. *JAMA Network Open.*

eMethods. Quality Metric Initiative, Natural Language Processing and Adherence

In 2015, Sutter Health/Palo Alto Medical Foundation (PAMF) implemented a quality improvement initiative to promote consistent monitoring of colonoscopy performance metrics among gastroenterologists. Each ambulatory surgery center (ASC) within PAMF conducts quarterly meetings to review multiple quality indicators, including, but not limited to procedure volume, cecal intubation rate, bowel preparation quality, withdrawal times, new equipment, complications, adenoma detection rate (ADR), and sessile serrated lesion detection rate (SSLDR).

To ensure accuracy and completeness, pathology findings from each colonoscopy are entered by the performing physicians into the quality reporting system. There was no independent review of pathology other than by the performing gastroenterologists. The investigators of this study were not involved with any part of the quality reporting process. These metrics were made readily accessible to all gastroenterologists through internal dashboards, providing transparency and fostering adherence to quality documentation, which incentivize accurate reporting of pathology findings.

To determine adherence rate for physician reporting, we performed a validation study using 2020 pathology reports. We first identified all pathology reports in 2020. Using a previously validated natural language processing (NLP) method,[1] we applied a rule-based NLP algorithm to identify and classify polyp histology from unstructured pathology reports. We previously validated this algorithm and found agreement for adenoma detection was 98.0% ($\kappa = 0.959$). Agreement by adenoma subtype was as follows: tubular 94.3% ($\kappa = 0.971$), tubulovillous 96.0% ($\kappa = 0.981$), villous 44.2% ($\kappa = 0.665$), and sessile serrated 96.0% ($\kappa = 0.980$).

Among the 10,520 pathology reports in 2020, NLP detected adenomas in 7253 reports in 2020, while physicians documented corresponding adenomas in 7512 cases. Since NLP is only 98% accurate in detecting adenoma, this likely underestimated the number of true adenomas in the pathology reports. Overall, this suggests that adenoma reporting by physicians is close to 100%.

Adherence Rate of Physician Reporting in 2020

Number of screening and surveillance colonoscopy in 2020	13,541
Number of colonoscopies with pathology reports	10,520
NLP with adenoma	7,253
Quality Reports of Adenoma by physician	7,521

eReference.

1. Liang SY, Oscarson B, Kenkare P, et al. Trends in Detection of Adenoma and Sessile Serrated Lesions Over a Decade in a Community-Based Healthcare System. Clin Transl Gastroenterol. 2024;15(3):e00683.

© 2026 Huang ES et al. *JAMA Network Open.*

eTable 1. SSLDR and ADR at Provider Level

	SSLDR		
	**Lowest Tertile (0.26-7.34%)**	**Middle Tertile (7.35-13.09%)**	**Highest Tertile (13.10-24.24%)**
Sample size, No.	16	17	17
Median ADR (%), [IQR]	31.2 (26.7, 43.6)	43.2 (38.6, 46.8)	52.0 (51.1, 56.5)
ADR, n (%)			
Lowest Tertile (12.40-37.65%)	14 (87.5%)	2 (11.8%)	0 (0%)
Middle Tertile (37.66-46.75%)	2 (12.5%)	11 (64.7%)	4 (23.5%)
Highest Tertile (47.76-61.73%)	0 (0%)	4 (23.5%)	13 (76.5%)

SSLDR: sessile serrated lesion detection rate; ADR: adenoma detection rate

© 2026 Huang ES et al. *JAMA Network Open.*

eTable 2. Risk of PCCRC According to SSLDR Quartiles Among Screening and Surveillance Colonoscopies (n=235,069)

	1st Quartile SSLDR	2nd Quartile SSLDR	3rd Quartile SSLDR	4th Quartile SSLDR	Continuous SSLDR[f]	*P*-trend (linear)
All PCCRC[a]						
No of cases/Person years	59/227682	102/460897	68/336322	65/291364	294/1316265	
Incidence Rate (Case/10,000 person-years)	2.59	2.21	2.02	2.23	2.23	
Age-adjusted HR (95% CI)	1.0	0.88 (0.63-1.24)	0.83 (0.57-1.20)	0.94 (0.64-1.38)	0.99 (0.97, 1.02)	0.56
Multivariate HR (95% CI)[b]	1.0	0.88 (0.62-1.25)	0.83 (0.57-1.20)	0.90 (0.61-1.32)	0.99 (0.96, 1.01)	0.32
Proximal PCCRC[c]						
No of cases/Person years	32/227682	36/460897	30/336322	21/291364	119/1316265	
Incidence Rate (Case/10,000 person-years)	1.41	0.78	0.89	0.72	0.90	
Age-adjusted HR (95% CI)	1.0	0.58 (0.35-0.98)	0.68 (0.39-1.16)	0.56 (0.31-1.03)	0.97 (0.93, 1.01)	0.16
Multivariate HR (95% CI)	1.0	0.63 (0.37-1.06)	0.71 (0.41-1.22)	0.58 (0.31-1.07)	0.97 (0.92, 1.01)	0.12
Distal PCCRC[d]						
No of cases/Person years	25/227682	62/460897	35/336322	41/291364	163/1316265	
Incidence Rate (Case/10,000 person-years)	1.10	1.35	1.04	1.41	1.24	
Age-adjusted HR (95% CI)	1.0	1.26 (0.76-2.06)	1.00 (0.58-1.71)	1.38 (0.80-2.38)	1.01 (0.98, 1.04)	0.56
Multivariate HR (95% CI)	1.0	1.18 (0.72-1.94)	0.96 (0.56-1.64)	1.26 (0.72-2.18)	1.01 (0.97, 1.04)	0.79
Advanced stage PCCRC[e]						
No of deaths/Person years	16/227682	29/460897	20/336322	19/291364	84/1316265	
Incidence Rate (Case/10,000 person-years)	0.70	0.63	0.59	0.65	0.64	
Age-adjusted HR (95% CI)	1.0	0.92 (0.48-1.76)	0.89 (0.44-1.77)	1.00 (0.48-2.11)	1.00 (0.95, 1.04)	0.86
Multivariate HR (95% CI)	1.0	0.95 (0.49-1.86)	0.90 (0.44-1.81)	1.00 (0.48-2.11)	0.99 (0.95, 1.04)	0.76

[a]Includes 25 colorectal cancer in an unknown location.

[b]Multivariate analysis included age (18-50, 50-75, >75 years of age), sex (female, male), BMI (<25, 25-30, >30 kg/m^2), race and ethnicity (African Americans, Asians, White, Other [American Indian or Alaska Native, Native Hawaiian or Other Pacific Islander, multi-race, other race and ethnicity, or race and ethnicity not otherwise specified]), smoking history (current smoker, former smoker, never smoker, unknown), CCI (0, ≥1), and removal of conventional adenoma (no, yes, unknown)

[c]Proximal PCCRC includes cancer in the appendix, cecum, ascending colon, hepatic flexure, transverse colon.

[d]Distal PCCRC includes cancer in the splenic flexure, descending colon, sigmoid colon, rectosigmoid colon and rectum.

[e]Advanced stage PCCRC includes stage 3 or 4 colorectal cancer.

[f] Multivariate analysis using same variables as above, but with continuous SSLDR.

PCCRC: postcolonoscopy colorectal cancer; SSLDR: sessile serrated lesion detection rate; HR: hazard ratio; CI: confidence interval

© 2026 Huang ES et al. *JAMA Network Open.*

eTable 3. Risk of Mortality According to SSLDR Quartiles Among Screening and Surveillance Colonoscopies (n=235,069)

	**1st Quartile SSLDR**	**2nd Quartile SSLDR**	**3rd Quartile SSLDR**	**4th Quartile SSLDR**	**Continuous SSLDR[b]**	***P*-trend (linear)[b]**
All-Cause Mortality						
No of deaths/Person years	1121/227853	2046/461302	1362/336543	1203/291549	5732/1317246	
Incidence Rate (Case/10,000 person-years)	49.2	44.4	40.5	41.3	43.5	
Age-adjusted HR (95% CI)	1.0	0.93 (0.86-1.01)	0.89 (0.82-0.97)	0.94 (0.86-1.02)	1.00 (0.99, 1.01)	0.96
Multivariate HR (95% CI)[a]	1.0	0.98 (0.90-1.06)	0.95 (0.87-1.04)	0.96 (0.88-1.05)	1.00 (0.99, 1.01)	0.65
PCCRC-related Mortality						
No of deaths/Person years	6/227853	10/461302	5/336543	4/291549	25/1317246	
Incidence Rate (Case/10,000 person-years)	0.26	0.22	0.15	0.14	0.19	
Age-adjusted HR (95% CI)	1.0	0.86 (0.30-2.43)	0.63 (0.19-2.07)	0.62 (0.18-2.17)	0.97 (0.90, 1.05)	0.48
Multivariate HR (95% CI)	1.0	0.87 (0.31-2.45)	0.66 (0.22-2.00)	0.60 (0.18-1.97)	0.98 (0.91, 1.04)	0.47

[a]Multivariate analysis included age (18-50, 50-75, >75 years of age), sex (female, male), BMI (<25, 25-30, >30 kg/m^2), race and ethnicity (African Americans, Asians, White, Other [American Indian or Alaska Native, Native Hawaiian or Other Pacific Islander, multi-race, other race and ethnicity, or race and ethnicity not otherwise specified]), smoking history (current smoker, former smoker, never smoker, unknown), CCI (0, ≥1), and removal of conventional adenoma (no, yes, unknown)

[b] Multivariate analysis using same variables as above, but with continuous SSLDR.

PCCRC: postcolonoscopy colorectal cancer; SSLDR: sessile serrated lesion detection rate; HR: hazard ratio; CI: confidence interval

© 2026 Huang ES et al. *JAMA Network Open.*

eTable 4. Risk of PCCRC According to SSLDR Quartiles Among Diagnostic Colonoscopy (n=93,347)

	**1st Quartile SSLDR**	**2nd Quartile SSLDR**	**3rd Quartile SSLDR**	**4th Quartile SSLDR**	**Continuous SSLDR[f]**	***P*-trend (linear)**
All PCCRC[a]						
No of cases/Person years	87/147678	72/231418	66/181260	43/162195	268/722551	
Incidence Rate (Case/10,000 person-years)	5.89	3.11	3.64	2.65	3.71	
Age-adjusted HR (95% CI)	1.0	0.58 (0.39-0.86)	0.66 (0.45-0.97)	0.49 (0.32-0.74)	0.96 (0.94, 0.99)	0.002
Multivariate HR (95% CI)[b]	1.0	0.63 (0.43-0.92)	0.69 (0.47-1.02)	0.53 (0.35-0.81)	0.96 (0.93, 0.98)	0.002
Proximal PCCRC[c]						
No of cases/Person years	45/147678	32/231418	27/181260	20/162195	124/722551	
Incidence Rate (Case/10,000 person-years)	3.05	1.38	1.49	1.23	1.72	
Age-adjusted HR (95% CI)	1.0	0.51 (0.27-0.99)	0.53 (0.29-0.95)	0.45 (0.24-0.84)	0.95 (0.91, 0.99)	0.02
Multivariate HR (95% CI)	1.0	0.57 (0.31-1.05)	0.53 (0.29-0.96)	0.47 (0.25-0.89)	0.95 (0.91, 0.99)	0.01
Distal PCCRC[d]						
No of cases/Person years	36/147678	39/231418	36/181260	20/162195	131/722551	
Incidence Rate (Case/10,000 person-years)	2.44	1.69	1.99	1.23	1.81	
Age-adjusted HR (95% CI)	1.0	0.73 (0.44-1.20)	0.86 (0.50-1.46)	0.54 (0.31-0.97)	0.97 (0.93, 1.00)	0.05
Multivariate HR (95% CI)	1.0	0.76 (0.45-1.28)	0.96 (0.56-1.64)	0.63 (0.35-1.16)	0.97 (0.94, 1.01)	0.16
Advanced stage PCCRC[e]						
No of deaths/Person years	25/147678	17/231418	22/181260	16/162195	80/722551	
Incidence Rate (Case/10,000 person-years)	1.69	0.73	1.21	0.99	1.11	
Age-adjusted HR (95% CI)	1.0	0.47 (0.24-0.92)	0.78 (0.41-1.51)	0.64 (0.33-1.27)	0.99 (0.95, 1.03)	0.54
Multivariate HR (95% CI)	1.0	0.49 (0.25-0.97)	0.77 (0.38-1.54)	0.64 (0.31-1.32)	0.98 (0.94, 1.03)	0.40

[a]Includes 25 colorectal cancer in an unknown location.

[b] Multivariate analysis included age (18-50, 50-75, >75 years of age), sex (female, male), BMI (<25, 25-30, >30 kg/m^2), race and ethnicity (African Americans, Asians, White, Other [American Indian or Alaska Native, Native Hawaiian or Other Pacific Islander, multi-race, other race and ethnicity, or race and ethnicity not otherwise specified]), smoking history (current smoker, former smoker, never smoker, unknown), CCI (0, ≥1), and removal of conventional adenoma (no, yes, unknown)

[c]Proximal PCCRC includes cancer in the appendix, cecum, ascending colon, hepatic flexure, transverse colon.

[d]Distal PCCRC includes cancer in the splenic flexure, descending colon, sigmoid colon, rectosigmoid colon and rectum.

[e]Advanced stage PCCRC includes stage 3 or 4 colorectal cancer.

[f]Multivariate analysis using same variables as above, but with continuous SSLDR.

PCCRC: postcolonoscopy colorectal cancer; SSLDR: sessile serrated lesion detection rate; HR: hazard ratio; CI: confidence interval

© 2026 Huang ES et al. *JAMA Network Open.*

eTable 5. Risk of Mortality According to SSLDR Quartiles Among Diagnostic Colonoscopy (n=93,347)

	1st Quartile SSLDR	2nd Quartile SSLDR	3rd Quartile SSLDR	4th Quartile SSLDR	Continuous SSLDR[b]	*P*-trend (linear)
All-Cause Mortality						
No of deaths/Person years	1674/148032	1950/231766	1641/181603	1468/162306	6733/723707	
Incidence Rate (Case/10,000 person-years)	113.1	84.1	90.4	90.4	93.0	
Age-adjusted HR (95% CI)	1.0	0.83 (0.77-0.90)	0.86 (0.80-0.93)	0.93 (0.86-1.004)	1.00 (0.99, 1.01)	0.70
Multivariate HR (95% CI)[a]	1.0	0.90 (0.84-0.97)	0.84 (0.78-0.91)	0.97 (0.90-1.06)	1.00 (0.99, 1.01)	0.81
PCCRC-related Mortality						
No of deaths/Person years	19/148032	5/231766	6/181603	9/162306	39/723707	
Incidence Rate (Case/10,000 person-years)	1.28	0.22	0.33	0.55	0.54	
Age-adjusted HR (95% CI)	1.0	0.18 (0.07-0.49)	0.27 (0.11-0.68)	0.53 (0.23-1.23)	0.96 (0.89, 1.03)	0.25
Multivariate HR (95% CI)	1.0	0.23 (0.08-0.65)	0.25 (0.10-0.65)	0.62 (0.26-1.51)	0.95 (0.88, 1.03)	0.21

[a]Multivariate analysis included age (18-50, 51-75, >75 years of age), sex (female, male), BMI (<25, 25-30, >30 kg/m^2), race and ethnicity (African Americans, Asians, White, Other [American Indian or Alaska Native, Native Hawaiian or Other Pacific Islander, multi-race, other race and ethnicity, or race and ethnicity not otherwise specified]), smoking history (current smoker, former smoker, never smoker, unknown), CCI (0, ≥1), and removal of conventional adenoma (no, yes, unknown)

[b]Multivariate analysis using same variables as above, but with continuous SSLDR

PCCRC: postcolonoscopy colorectal cancer; SSLDR: sessile serrated lesion detection rate; HR: hazard ratio; CI: confidence interval

© 2026 Huang ES et al. *JAMA Network Open.*

eTable 6. Baseline Characteristics With Income

Characteristics	Overall[a] (n=328,416)	Sessile Serrated Lesions Detection Rate (SSLDR)			
		1st Quartile 0.26-6.4% (n=45,869)	2nd Quartile 6.5-10.3% (n=119,056)	3rd Quartile 10.4-14.6% (n=79,240)	4th Quartile 14.7-24.2% (n=84,251)
Income[b] ($), median, [IQR]	93,787 (70,934-117,366)	92,084 (79,644-113,537)	99,390 (85,270-121,527)	90,747 (77,105-116,091)	87,779 (68,791-114,870)
Income (tertiles, $), No. (%)					
Low (90-87465)	108,198 (32.9)	18,988 (33.8)	25,709 (23.6)	31,314 (36.7)	32,187 (41.1)
Middle (87,465-105,934)	108,066 (32.9)	19,401 (34.5)	41,114 (37.8)	26,748 (31.4)	20,803 (26.6)
High (105,935-219,554)	106,730 (32.5)	16,845 (30)	40,310 (37.1)	25,807 (30.3)	23,768 (30.4)
Unknown	5,422 (1.7)	959 (1.7)	1,599 (1.5)	1,361 (1.6)	1,503 (1.9)

[a] 328,416 colonoscopies amongst 226,695 unique patients

[b]Median household income data at zip code level linked to geocoded current address of patient population

© 2026 Huang ES et al. *JAMA Network Open.*

eTable 7. Risk of Mortality According to SSLDR Quartiles Adjusting for Income

	1st Quartile SSLDR	2nd Quartile SSLDR	3rd Quartile SSLDR	4th Quartile SSLDR	Continuous SSLDR[b]	*P*-trend (linear)
All-Cause Mortality						
No of deaths/Person years	2795/3754885	3996/693068	3003/518147	2671/453854	12465/2040953	
Incidence Rate (Case/10,000 person-years)	74.4	57.7	58.0	58.9	61.07	
Age-adjusted HR (95% CI)	1.0	0.83 (0.78-0.88)	0.84 (0.79-0.89)	0.91 (0.85-0.97)	1.00 (1.00-1.00)	0.88
Multivariate HR (95% CI)[a]	1.0	0.94 (0.88-0.99)	0.89 (0.83-0.94)	0.97 (0.91-1.04)	1.00 (1.00-1.00)	0.89
PCCRC-related Mortality						
No of deaths/Person years	25/3754885	15/693068	11/518147	13/453854	64/2040953	
Incidence Rate (Case/10,000 person-years)	0.67	0.22	0.21	0.29	0.31	
Age-adjusted HR (95% CI)	1.0	0.35 (0.17-0.71)	0.35 (0.17-0.72)	0.59 (0.25-1.12)	0.96 (0.92-1.02)	0.20
Multivariate HR (95% CI)	1.0	0.42 (0.21-0.85)	0.36 (0.17-0.74)	0.59 (0.28-1.26)	0.96 (0.91-1.02)	0.21

[a]Multivariate analysis included age (18-50, 51-75, >75 years of age), sex (female, male), BMI (<25, 25-30, >30 kg/m^2), race and ethnicity (African Americans, Asians, White, Other [American Indian or Alaska Native, Native Hawaiian or Other Pacific Islander, multi-race, other race and ethnicity, or race and ethnicity not otherwise specified]), smoking history (current smoker, former smoker, never smoker, unknown), CCI (0, ≥1), indication of colonoscopy (diagnostic, screening/surveillance), removal of conventional adenoma (no, yes, unknown), and income (low, middle, high, unknown)

[b]Multivariate analysis using same variables as above, but with continuous SSLDR

PCCRC: postcolonoscopy colorectal cancer; SSLDR: sessile serrated lesion detection rate; HR: hazard ratio; CI: confidence interval

© 2026 Huang ES et al. *JAMA Network Open.*
