# Supplementary material for: Sessile Serrated Lesion Detection Rate and Colorectal Cancer Risk and Mortality
Source: JAMA Netw Open. 2026 Feb 17;9(2):e2556964. doi: 10.1001/jamanetworkopen.2025.56964 (PMC12914494; doi:10.1001/jamanetworkopen.2025.56964)
Supplement: Supplement 2. — Data Sharing Statement [file jamanetwopen-e2556964-s002.pdf]

Data Sharing Statement

Huang. Sessile Serrated Lesion Detection Rate and Colorectal Cancer Risk and Mortality. *JAMA Netw Open*. Published February 17, 2026. doi:10.1001/jamanetworkopen.2025.56964

Data
Data available: No

Additional Information
Explanation for why data not available: There is patient confidential information and will require IRB approval to allow data access.
